# Selection of chemical markers for the quality control of medicinal plants of the genus *Cecropia*

**DOI:** 10.1080/13880209.2017.1307421

**Published:** 2017-04-04

**Authors:** Andrés Rivera-Mondragón, Orlando O. Ortíz, Sebastiaan Bijttebier, Arnold Vlietinck, Sandra Apers, Luc Pieters, Catherina Caballero-George

**Affiliations:** a Natural Products & Food Research and Analysis (NatuRA), Department of Pharmaceutical Sciences, University of Antwerp, Antwerp, Belgium;; b Herbarium PMA, Universidad de Panamá, Estafeta Universitaria, Panama City, Republic of Panama;; c Group of Pharmaceutical Research, Institute of Scientific Research and High Technology Services (INDICASAT-AIP), Panama, Republic of Panama

**Keywords:** Urticaceae, herbal medicine, quality control

## Abstract

**Context:** Several *Cecropia* (Cecropiaceae) species are traditionally used in Latin America for the treatment of a variety of diseases including diabetes, arterial hypertension, asthma, bronchitis, anxiety, and inflammation. At present, a number of commercial products based on these plants have been introduced into the market with very little information on methods for guaranteeing their quality and safety.

**Objective:** This work proposes potential chemical markers for the quality control of the raw materials of *Cecropia obtusifolia* Bertol., *Cecropia peltata* L., *Cecropia glaziovii* Snethl., *Cecropia pachystachya* Trécul, and *Cecropia hololeuca* Miq.

**Methods:** The Herbal Chemical Marker Ranking System (Herb MaRS) developed by the National Institute of Complementary Medicine (NICM) at the University of Western Sydney was used for selecting chemical markers for the quality control of selected medicinal species of *Cecropia*. This review covers the period from 1982 to 2016.

**Results:** Chlorogenic acid, flavonoidal glycosides (orientin, isoorientin, vitexin, isovitexin, and rutin), catechin, epicatechin, procyanidins (B2, B5, and C1), steroids (β-sitosterol), and triterpenoids (α-amyrin, pomolic, tormentic and ursolic acids) were selected as chemical markers for the quality control of the leaves.

**Conclusion:** It is necessary to establish comprehensive standards for guaranteeing quality, safety and efficacy of herbal drugs. The selection of adequate chemical markers for quality control purposes requires a good knowledge about the chemical composition of medicinal plants and their associated biological properties. To the best of our knowledge this review article is the first to address the identification and quantitative determination of the chemical markers for the genus *Cecropia*.

## Introduction

Herbal medicines, also called botanical medicines or phytomedicines, are described as any form of plant or plant product used in the maintenance of health as well as in the prevention, improvement, diagnosis or treatment of diseases. These products include herbs (leaves, flowers, fruits, seeds, stems, woods, barks, roots, or other plant parts), herbal material (fresh juices, gums, essential oils, and resins), herbal preparations (extracts, tinctures, and fatty oils from herbal materials) and finished herbal products (World Health Organization [WHO] 2000).

The WHO estimates that approximately 80% of people around the world have used herbal medicines and emphasizes that these products play an important role in health care systems (Montoro et al. 2012). Therefore, it is essential to establish guidelines for assessing their quality (World Health Organization [WHO] 2011).

Despite the fact that herbal medicines are popularly consumed and are worldwide recognized as safe, several adverse reactions have been associated with their use. In fact, their variable composition, the presence of toxic contaminants, pesticides, microbial contaminants, and adulteration with other plant species or synthetic drugs can affect their quality, efficacy, and safety (Chan 2003).

Herbal medicinal products usually contain complex mixtures of active chemicals, thus the selection of characteristic chemical constituents for analytical testing is useful for guaranteeing and demonstrating adequate and consistent quality (European Medicines Agency [EMA] 2008). Nowadays, a number of commercial products based on medicinal plants have been introduced into the market with still very little information about their chemical constituents. Plants of the genus *Cecropia* (Urticaceae) are a representative example of this situation.

In general terms, the genus *Cecropia* is characterized as a dioecious tree, few-branched, usually with a candelabrum-like branching system, a hollow trunk, sometimes with stilt roots, fully amplexicaul stipules, peltate blades with one to two trichilia at the base of the petioles, inflorescences arranged in digitate clusters (or a single inflorescence), usually enveloped by a spathe until anthesis, interfloral bracts absent, flowers with two stamens, and small, dry fruits enveloped by a tubular greenish perianth (Berg et al. 1990; Berg & Roselli 2005). This genus is a widespread and abundant fast-growing tree distributed across the tropical and subtropical rainforest from Mexico to Central and South America below 2600 m above sea level (Franco-Rosselli & Berg 1997). The plants of this genus comprise 61 species (Berg & Roselli 2005) and are popularly known as ‘yarumo’, ‘guarumo’, ‘guarumbo’, ‘embaúba’, ‘ambay’, ‘torém’, ‘trumpet tree’, among other folk names (Luengas-Caicedo et al. 2007; Costa et al. 2011a; Montoya Peláez et al. 2013; Ospina Chávez et al. 2013).

*Cecropia* species have a relevant ecological significance; due to their rapid growth rate, they are primary colonizers of deforested tropical areas (Monro 2009) and act as invasive species in non-native regions (Conn et al. 2012; Global Invasive Species Database [GISD] 2016). Most *Cecropia* species are ant-plants or myrmecophytes, that is, they live in a mutualistic relationship with a colony of symbiotic ants (especially the genus *Azteca*). They possess specialized structures for offering shelter, food, and space to inhabit for ants in exchange for protection against natural enemies (Dejean et al. 2010; Oliveira et al. 2015).

This review contains a compilation of chemical markers of the most extensively studies species of the *Cecropia* genus, which have been used in popular medicine of many Latin American countries. This information can be useful for the development of monographs in pharmacopoeias or by the pharmaceutical industry to implement suitable criteria on their quality, avoiding adulterated or counterfeit herbal products derived from some species of the genus *Cecropia*.

### Traditional use of the genus *Cecropia*

The biological importance of this genus is associated with its multiple medicinal claims in several Latin American countries. Five medicinal species within this genus selected for this work are widely used as diuretics, antioxidants, antitussives, expectorants and for the treatment of several diseases such as cough, asthma, hypertension, diabetes, inflammation and central nervous system disorders (anxiety and depression) (Costa et al. 2011a; Gazal et al. 2014; Pacheco et al. 2014). In addition, they have been reported for their wound healing, analgesic and antimicrobial properties (Souccar et al. 2008). The range of therapeutic properties attributed to these plants has been correlated to their content of flavonoids, proanthocyanidins (Luengas-Caicedo et al. 2007), terpenoids, steroids (Ospina Chávez et al. 2013), chlorogenic and caffeic acid (Müller et al. 2016), and other phenolic compounds (Gazal et al. 2014).

### *Cecropia obtusifolia* Bertol

In folk medicine the dried leaves of *C. obtusifolia* are used as an infusion primarily for the treatment of diabetes and as an anti-inflammatory agent (Pérez-Guerrero et al. 2001). The aqueous infusion is prepared using 15 g of dry leaves boiled in 500 mL of water. The resulting cold infusion is drunk over the course of the day (Andrade-Cetto & Vázquez 2010). In Mexico, the leaves, stem, bark, and root are widely used for the empirical treatment of diabetes type 2 (Farmacopea Herbolaria de los Estados Unidos Mexicanos [FHEUM] 2001; Revilla-Monsalve et al. 2007; Alonso-Castro et al. 2008; Aarland et al. 2015). This plant was included in the Herbal Pharmacopeia of the United Mexican States (Farmacopea Herbolaria de los Estados Unidos Mexicanos [FHEUM] 2001). In addition, decoctions of the leaves of this plant are used in El Salvador as a sedative and for the treatment of arthritis and rheumatism (Pérez-Guerrero et al. 2001). In Costa Rica, this plant is popularly used for the treatment of arterial hypertension and as a diuretic agent (Farmacopea Herbolaria de los Estados Unidos Mexicanos [FHEUM] 2001). *Cecropia obtusifolia* is also traditionally used in Latin America to treat heart failure, cough, asthma, bronchitis, fever, hepatic and kidney disorders, wounds, ant and scorpion stings (Farmacopea Herbolaria de los Estados Unidos Mexicanos [FHEUM] 2001; Guerrero et al. 2010).

### *Cecropia peltata* L

The leaves of *C. peltata* are traditionally used as an infusion to treat cardiovascular, metabolic, and respiratory disorders, for their wound-healing and diuretic effects (Nayak 2006; Ospina Chávez et al. 2013). This infusion is prepared in a similar way as *C. obtusifolia* (Andrade-Cetto & Vázquez 2010). In Mexico, Brazil, and Trinidad and Tobago the leaves are taken to treat diabetes mellitus (Andrade-Cetto & Heinrich 2005; Nicasio et al. 2005; Lans 2006; Agra et al. 2007; Andrade-Cetto & Vázquez 2010). In Brazil and Trinidad and Tobago they are used for treating heart diseases and hypertension (Lans 2006; Agra et al. 2007). In Colombia it is used as a sedative and antimicrobial agent (Rojas et al. 2006; Ospina Chávez et al. 2013). In French Guinea, the infusion is used to treat albuminuria, kidney infections, heart conditions and nervous diseases, and to promote good kidney function (DeFilipps et al. 2004).

### *Cecropia glaziovii* Snethl

*Cecropia glaziovii* is reputed in Latin American folk medicine to treat heart, inflammatory and respiratory conditions (Souccar et al. 2008). In Brazil, the extract of the leaves is traditionally used as antidiabetic, anti-inflammatory and anti-hypertensive agent (Müller et al. 2016), and for the treatment of cough and bronchitis (Lima-Landman et al. 2007). The Brazilian Ministry of Health (CEME Program, 1984–1998) selected this genus as a prototype to develop medicinal plants useful in public health. Furthermore, the Brazilian Pharmaceutical Formularies included it to treat heart failure, cough, bronchitis, dyspnea, and asthma (Tanae et al. 2007).

### *Cecropia pachystachya* Trécul (Syn.: *Cecropia adenopus* Mart. ex Miq., *Cecropia lyratiloba* Miq. and *Cecropia catarinensis* Cuatrec.)

*Cecropia pachystachya* grows in the forest of the neotropical region of South America, and is known as embaúba and ambay in Brazil and Argentina, respectively (Consolini et al. 2006). The leaves and bark of this plant are popularly used as antitussive, expectorant, antiasthmatic, diuretic and hypoglycemic agent, for relieving inflammation, wound healing, hypertension, cardiac diseases, and as antipyretic for the treatment of fever in malaria as well (Ramos Almeida et al. 2006; Uchoa et al. 2010; Gazal et al. 2014). In Argentina, the aqueous extract is prepared by boiling 40 g of dried leaves in 1 L of water at a dosage of three cups (200 mL each) a day. In addition, it was incorporated in the Argentinian National Pharmacopoeia (VI Ed. 1978), which recommends the medicinal use of this plant as a 5% decoction (Consolini & Migliori 2005; Costa et al. 2011b).

### *Cecropia hololeuca* Miq

Ethnomedical indications for *C. hololeuca* include diuretic, antihypertensive, sedative, anti-inflammatory, expectorant antiasthmatic, cough suppressant, anti-thermal, and anticancer agent (Ramos Almeida et al. 2006; Botsaris 2007; Hernández Carvajal & Luengas Caicedo 2013). In Brazil, the maceration or decoction of a handful of leaves or roots in a litre of water is used as diabetic and diuretic (Agra et al. 2007). Leaves, fruits and sprout juices are traditionally indicated as adjuvant in malaria with very high fever or neurological symptoms in the same country (Botsaris 2007). The syrup of *C. hololeuca* was incorporated in the First Edition of Brazilian Pharmacopeia in 1929 (Petenatti et al. 1998; Tanae et al. 2007; Costa et al. 2011b).

## Biological activities and chemical compounds reported in the genus *Cecropia*

### *Cecropia obtusifolia* Bertol

Its hypoglycaemic activity has been demonstrated by a number of experimental designs. The intravenous administration of an aqueous extract (AE) from leaves induced a significant reduction of blood glucose level in normal (33%) and pancreatectomized (46%) dogs with respect to the control group. This effect was not related to a stimulus for insulin secretion (Mellado & Lozoya 1984). Besides, AE and butanolic (BuE) extracts prepared from leaves were able to inhibit gluconeogenesis *in vivo* by a pyruvate tolerance test in streptozotozin (n5-STZ) induced diabetic rats (Andrade-Cetto & Wiedenfeld 2001; Andrade-Cetto & Vázquez 2010) and to reduce the glucose-6-phosphatase activity *in vitro* from rat liver microsomes with IC_50_ of 224 μg/mL (AE) and 160 μg/mL (BuE) (Andrade-Cetto & Vázquez 2010). BuE demonstrated an inhibition of α-glycosidase activity *in vitro* in a degree greater than acarbose with an IC_50_ of 14 μg/mL (Andrade-Cetto et al. 2008). In the same way, methanolic extracts (ME) of leaves produce a significant reduction (33.3–35.7%) of plasma glucose level in healthy mice (Nicasio et al. 2005).

A significant and sustained hypoglycaemic effect of AE from leaves has been shown in type 2 diabetic patients after a clinical trial of 32 weeks of treatment. A significant reduction of glycosylated haemoglobin (HbA1c) was reported after 6 weeks. No significant changes in patient’s insulin secretion, or alanine aminotransferase (ALT), aspartate aminotransferase (AST), and alkaline phosphatase (ALKP) levels were found; this indicates a low hepatotoxicity during the treatment (Revilla-Monsalve et al. 2007). In another study, infusion of *C. obtusifolia* as an adjunct treatment of glibenclamide (on patients with non-optimal response) produced a substantial hypoglycaemic effect (15.25%) and a great efficacy for reducing the fasting blood glucose (36.6%) (Herrera-Arellano et al. 2004).

Chlorogenic acid and isoorientin, the main compounds found in BuE and AE, have exhibited a hypoglycaemic effect in streptozotozin diabetic rats (Andrade-Cetto & Wiedenfeld 2001; Andrade-Cetto et al. 2008). The chlorogenic acid activity by stimulating the glucose uptake in both insulin-sensitive and insulin-resistant adipocytes was comparable with the antidiabetic drug rosiglitazone (Alonso-Castro et al. 2008). Furthermore, the compound was identified as a specific inhibitor of glucose-6-phosphate translocase component (Gl-6-P translocase) in microsomes of rat liver (Andrade-Cetto & Heinrich 2005). Besides this, the potent antioxidant effect of isoorientin, which has been reported to contribute to the hypoglycaemic effect of chlorogenic acid, may explain and support the medicinal use of *C. obtusifolia* as antidiabetic (Andrade-Cetto & Heinrich 2005).

Some pharmacological experiments revealed its anti-hypertensive effects. A lyophilized AE from leaves produced a fall in arterial pressure (−23.5% relative to preinjection values) when it was administered to hypertensive conscious rats (Salas et al. 1987). Additionally, an important blood pressure-lowering effect of ethanol extract (EtE) from leaves was determined in anesthetized male rats (Vidrio et al. 1982). On the other hand, a preliminary biological screening by radioligand-binding techniques demonstrated that both MeOH/CH2Cl2 (1:1) and EtE extracts from stem and leaves at 100 μg/mL had a higher inhibition than 50% of Angiotensin II receptor type 1 (AT_1_) and Endothelin receptor A (ET_A_) (Caballero-George et al. 2001).

Other biological activities have been reported, such as a central depressor effect (Hole-board, traction, evasion, and rota-rod tests), peripheral analgesic effect (acetic and formalin test), and as a topical and systemic anti-inflammatory agent (carrageenan-induced oedema) (Pérez-Guerrero et al. 2001). This last activity could justify its traditional use in rheumatic and kidney inflammation pathologies. Active compounds with anti-inflammatory and anti-hypertensive activities have not been clearly identified as yet.

AE from leaves of *C. obtusifolia* have shown a low toxicity profile in different experimental models. No statistically significant increases in cytotoxicity and/or genotoxicity was found in the wing somatic mutation and recombination test (SMART) in flies and in the human micronucleus assay from lymphocytes obtained from type 2 diabetes patients (Toledo et al. 2008). On the other hand, the acute toxicity test in mice reported a median lethal dose (LD_50_) of 1450 ± 70 mg/kg animal (11.21 ± 0.52 g of plant/kg of weight) (Pérez-Guerrero et al. 2001).

Nineteen additional compounds have been identified from leaves of *C. obtusifolia*. Five anthraquinones (aloe-emodin, emodin, rhein, chrysophanol, and physcion) (Yan et al. 2013), two saturated fatty acids (palmitic and stearic acid) (Guerrero et al. 2010), five steroids [β-sitosterol, stigmasterol (Andrade-Cetto & Heinrich 2005), stigmast-4-en-3-one, 4-cholestene-3,24-dione, and 4,22-cholestadien-3-one (Guerrero et al. 2010)], and vanillic acid (Guerrero et al. 2010). There are several other reports that have proven their anti-inflammatory activity by suppressing activation inflammatory pathways in different *in vitro* and *in vivo* systems (Gabay et al. 2010; Loizou et al. 2010; Pan et al. 2010; Tewtrakul et al. 2010; Kim et al. 2011; Aparna et al. 2012; Choi et al. 2013; Kshirsagar et al. 2014; Park et al. 2016). In addition, the hypoglycaemic effect of stigmast-4-en-3-one has been claimed as well (Jamaluddin et al. 1995; Alexander-Lindo et al. 2004). Besides these, 4-vinyl-2-methoxy-phenol, 2-methylbenzaldehyde, 2,3-dihydrobenzofuran, 3′-methoxyacetophenone (Guerrero et al. 2010), 1-(2-methyl-1-nonen-8-il)-aziridine, and 4-ethyl-5-(n-3-valeroil)-6-hexahydrocoumarin (Andrade-Cetto & Heinrich 2005) have also been reported. All these compounds are shown not to be related to the traditional use of these plants and, moreover, are not commercially available. Active compounds with anti-hypertensive activities have not been clearly identified as yet.

### *Cecropia peltata* L

The hypoglycaemic effect of AE and BuE from leaves was demonstrated by the inhibition of gluconeogenesis *in vivo* with a pyruvate tolerance test in n5-STZ diabetic rat model (Andrade-Cetto et al. 2007; Andrade-Cetto & Vázquez 2010). Likewise, the reduction of glucose-6-phosphatase (obtained from rat liver microsomes) activity *in vitro* with an IC_50_ of 146 μg/mL (AE) and 150 μg/mL (BuE) was also reported (Andrade-Cetto & Vázquez 2010). Beside this, a reduction of plasma glucose level (58%) in healthy mice was observed after oral administration of ME from leaves (Nicasio et al. 2005). This activity was correlated with the relative high concentrations of chlorogenic acid and isoorientin in the extract (Andrade-Cetto et al. 2007).

The cardiotonic activity of AE from leaves was proved by the increase of contractility (positive-inotropic effect) on isolated guinea pig atria, but was also reported to cause injury to cardiomyocytes (Bipat et al. 2016). Pharmacological studies support the traditional use of *C. peltata* as wound healing agent. AE and EtE from leaves of this species showed a significant reduction of wound areas after topical and oral administration in a rat model (Nayak 2006). The chemical identity of the bioactive compounds was not clearly elucidated. In another study, an EtE showed antimicrobial activity by a high percentage of relative inhibition zone diameters against *Staphylococcus aureus* (78.0 ± 0.6)*, Bacillus cereus* (83.0 ± 0.3), and *Escherichia coli* (104.9 ± 0.0). It is suggested that the presence of steroids and amino acids could be responsible for this effect (Rojas et al. 2006). This correlation, however, is not scientifically confirmed.

### *Cecropia glaziovii* Snethl

Parallel studies have been performed in order to validate its traditional use as anti-inflammatory, anti-hypertensive, anti-asthmatic agent, for the treatment of gastric ulcers, bronchitis, anxiety, and depression. In one study, oral administration of AE of *C. glaziovii* showed a potent *in vivo* anti-inflammatory activity by a significant reduction in nitrite/nitrate concentrations, leucocytes migration, TNF-α, and IL-1β in the pleural cavity in a carrageenan-induced pleurisy rat model. Chlorogenic acid, isoorientin, and isovitexin were identified as the major compounds of this extract (Müller et al. 2016).

According to two independent investigations, oral administration of a standardized AE and its *n*-butanol fraction (BuF) from leaves reduced hypertension in normotensive, spontaneous and induced (by l-NAME and constriction of one renal artery) hypertensive rats. BuF produced inhibition of the pressor responses to noradrenaline, angiotensin I, and angiotensin II by 40% (Lapa et al. 1999; Lima-Landman et al. 2007). This effect was not correlated to angiotensin-converting enzyme activity (ACE) inhibition, increase of nitric oxide (NO) synthesis, or specific blockade of α1 and AT1 receptors. Even though the mechanism is unknown, it is suggested that BuF interferes with the calcium pathway in smooth muscle cells and neurons (Lapa et al. 1999; Lima-Landman et al. 2007). Moreover, a significant *in vitro* ACE-inhibition (91 ± 9%) was obtained with the ME from stipules of this plant. The active compounds were identified as catechin (16 ± 3%), epicatechin (34 ± 1%), isoquercitrin (32 ± 2%), isoorientin (48 ± 1%), procyanidin B_2_ (25 ± 5%), and C_1_(45 ± 2%) (Lacaille-Dubois et al. 2001).

An anti-asthmatic effect has been also reported. The administration of AE and BuF increased the concentration of histamine necessary to produce bronchospasm in guinea pigs by 5- and 2-fold, respectively. In addition, the maximal response of tracheal muscle to histamine was decreased by 13–55% after the administration of BuF. This effect appears to be related to a β-adrenergic activity (Delarcina et al. 2007).

On the other hand, BuF and its purified constituents reduced the immobility of rats in the forced swimming test (FST) pointing out an antidepressant-like effect. This activity was evidenced by the inhibition of serotonine uptake, dopamine, and noradrenalin in different brain regions in rats. The most active compounds *in vitro* were identified as catechin (IC_50_ 47.7, 7.9, 4.6 μg/mL), procyanidin B2 (IC_50_ 249.0, 24.3, 15.4 μg/mL), and procyandin B3 isomer (IC_50_ 117.5, 39.0, 18.5 μg/mL) (Rocha et al. 2007).

Another study reports the anti-ulcer and anti-acid effect of AE, BuF and its isolated compounds involved in the pharmacologic mechanism. The administration of BuF produced a decrease in the volume (28%) and total acidity (33%) of gastric secretion; and a reduction of total acidity of the histamine and bethanechol-induced gastric secretion (by 45% and 65%, respectively) in pylorus-ligated mice. Pretreatment of acute gastric mucosal lesion in a rat model with BuF reduced the index of mucosal damage (IMD) and the number of ulcers by 47 and 54%, respectively. All isolated compounds (isoorientin, orientin, isovitexin, catechin, epicatechin, and procyanidin B2, B3, B5, and C1) inhibited the rabbit gastric H^+^, K^+^ ATPase enzyme activity with IC_50_ values similar to that obtained with the original BuF (58.8 μg/mL) (Souccar et al. 2008).

The hepatoprotective and antiviral activity against Herpes simplex virus type 1 (HSV-1) was also investigated. EtE from leaves treatment attenuated CCl_4_-induced liver injury by AST and ALT activities, hepatic lipid peroxidation. This extract was also active against HSV-1 (acyclovir-resistant strain) by inhibiting its replication with EC_50_ = 40 μg/mL and selective index (SI) = 50 (Petronilho et al. 2012). In addition, low acute and chronic toxicity on pregnant rats, normal morphological development of rat offspring, no effects on fetal parameters and a LD_50_ higher than 5.0 g/kg were observed after the oral administration of AE in two different studies during the two last segments of the reproductive cycle (Gerenutti et al. 2008; Randazzo-Moura 2011).

### *Cecropia pachystachya* Trécul

The hypoglycaemic activity of the ME from leaves demonstrated a significant blood glucose reduction in glucose loading (68%, after 12 h) and induced-diabetic rats (60%, max. value). The extract showed a potent antioxidant effect with IC_50_ 3.1 μg/mL (DPPH assay) and EC_50_ 10.8 μg/mL (reduction power). The hypoglycaemic effect was attributed to its content of chlorogenic acid, isoorientin, and orientin as it has been reported before in species of this genus (Aragão et al. 2010).

The anti-inflammatory effect of *C. pachystachya* has been validated in different studies. β-Sitosterol isolated from the hexane extract (HE) of leaves was reported to have a significant anti-inflammatory capacity in the carrageenan-induced mouse pedal oedema assay (Hikawczuk et al. 1998). Likewise, pomolic acid isolated from the dichloromethane extract (DCME) of the leaves exhibited the ability to limit the inflammatory response in carrageenan-induced mouse paw oedema by 34–37%. Moreover, this compound reduced the *in vivo* production of IL-1β (39%) by inhibiting the viability of neutrophils through apoptosis (Schinella et al. 2008). Trans-phytol, α-amyrin and ursolic acid (identified from DCME) have been reported to exhibit anti-inflammatory effects (Schinella et al. 2008).

Furthermore, the ME of leaves produced a significant inhibition of oedema in acute ear mouse oedema induced by several agents, after topical (83%) and oral (52%) treatment. These effects were similar to that of indomethacin and dexamethasone (Aragão et al. 2013; Pacheco et al. 2014) and could be correlated with the high relative concentration and antioxidant properties of orientin, isoorientin, and chlorogenic acid quantified in the extract (Pacheco et al. 2014).

The potential use of this plant in the treatment of renal chronic diseases was demonstrated by reducing the inflammation and renal lesions in male Wistar rats submitted to 5/6 nephrectomy. These effects were associated with ACE-inhibition (67%), reduction of macrophage (ED-1 positive cells) infiltration, angiotensin II (AII) and c-Jun N-terminal kinase (p-JNK) expression, and arginase activity in renal cortex of rats. Chlorogenic acid and orientin were the main compounds identified in AE and EtE of leaves, which have been previously shown to have an anti-inflammatory effect (Maquiaveli et al. 2014).

Different studies have validated its neurological effects. An enriched C-glycosyl flavonoid fraction (EFF) and AE from leaves had antidepressant-like effects in mice and in male Wistar rats subjected to chronic mild and chronic unpredictable stress. Both extracts significantly reduced the immobility time in the forced swimming test (FST). This activity was correlated to the reduction and prevention of oxidative damage (decreased oxidative markers, increased activity of antioxidant enzymes, and prevented mitochondrial dysfunction) produced by the main compounds of EFF and AE (chlorogenic acid, isoorientin, orientin, isovitexin, and isoquercitrin) (Gazal et al. 2014; Ortmann et al. 2016).

Further research has demonstrated the potential use of the AE of leaves as an agent for preventing intervention during manic phases of bipolar disorders by reducing episode relapse and the oxidation damage associated. Pretreatment of female Wistar with AE for 14 days prevented hyperlocomotion and oxidative damage in the prefrontal cortex and hippocampus induced by ketamine. The HPLC profile of AE showed the presence of chlorogenic acid, orientin, isoorientin, isovitexin, and isoquercitrin. The antioxidant activity of these flavonoids is correlated to the antidepressant effect and it is believed to be able to prevent neurodegeneration in animal model as in clinical conditions as well (Gazal et al. 2015).

This plant has also been reported for its cardiovascular effects. AE of leaves showed hypotensive (decreased blood pressure until 46.2% of basal) and cardiotonic (increased heart rate until 133% of basal) activities on Wistar rats (Consolini & Migliori 2005). These results may be explained by central blockage of sympathetic nerves of vessels and central cholinergic inhibition of the heart, respectively. In this study the activities were not associated to specific compounds. Furthermore, a flavonoid fraction (FF) of ME of leaves induced cardiac depression (reduction to 56.7 ± 5.1%) and inhibited adrenaline-induced contractions of the aorta (34.2 ± 6.9%) in Wistar rats (Oliveira et al. 2003; Ramos Almeida et al. 2006). It is suggested that the vasodilation produced is the result of an endothelin-dependent effect probably by the stimulation of NO production. Orientin, isoorientin, isovitexin, and apigenin-6-galactosyl-6′′-*O*-galactopyranoside, the main flavonoids isolated from FF, did not seem to be active when tested individually

A preclinical study validated the folk use of wound healing of *C. pachystachya*. This study demonstrated that the gels containing ethyl acetate extract of leaves 2% and 5% promotes the healing process using the excision skin model in Wistar rat (Duque et al. 2016). The chemical fingerprint of the extract revealed the presence of chlorogenic acid, orientin, and isoorientin as its main compounds. The healing properties could be attributed to these compounds, since the high reactivity of the hydroxyl group of flavonoids inactivate free radicals and inhibit the oxidation of lipoproteins, promote diffusion of oxygen, increase of lymphatic drainage, reduce of oedema, and increase collagen synthesis (Duque et al. 2016).

In more recent investigations additional pharmacological properties have been reported for this plant, including anti-parasitic, antimicrobial, and anti-leukemic activities. EtE of wood, root, and leave reduced parasitaemia of malaria-infected mice (33–66%) and tormentic acid isolated from HE from root-wood was active against the W2 strain of *P. falciparum* (IC_50_ 11–15 μg/mL) (Uchoa et al. 2010). On the other hand, the isolated compounds from the ethyl acetate fraction of EtE of leaves demonstrated leishmanicidal properties by inhibition of *L. amazonensis* promastigotes arginase activity (Cruz et al. 2013). Chlorogenic acid, catechin, epicatechin, and isoquercitrin exhibited inhibition above 50% at 20 μM. Orientin, the most active compound, showed an IC_50_ of 7 μM. Apigenin, luteolin, and quercetin were also identified and reported as inhibitors of arginase. No significant cell toxicity was shown in cultures of splenocytes (Cruz et al. 2013). In addition, C-glycosyl flavonoids (orientin, isoorientin, vitexin, and isovitexin), rutin, and chlorogenic acid (all isolated from the AE of leaves), have been reported for the first time as quorum sensing (QS) inhibitors using *C. violaceum* (inhibition of violacein pigment) and *E. coli* (bioluminescent inhibition) as biosensors in the agar diffusion tests (Brango-Vanegas et al. 2014).

Moreover, eight isolated triterpenes from the roots were identified as tormentic, 2-α-acetyl tormentic, 3-β-acetyl tormentic, euscaphic, 2-*O*-acetyl euscaphic, isoarjunolic acids, 2-α-acetoxy-3β,19α-dihydroxy-11α,12α-epoxy-ursan-28,13β-olide, and 3-β-acetoxy-2α,19α-dihydroxy-11α,12α-epoxy-ursan-28,13β-olide (Oliveira et al. 2005; Machado et al. 2008; Rocha et al. 2007, 2012a). The first four products were shown to be cytotoxic against sensitive and multidrug resistant leukaemia cell lines (respective IC_50_ values in parenthesis): K562 (76.71, 89.36, 38.35, 56.61 μM) and vincristine-resistant human erythromyeloblastoid leukemia (Lucena-1; 83.79, 80.25, 41.38, 72.87 μM) (Rocha et al. 2007, 2012a, 2012b).

### *Cecropia hololeuca* Miq

The EtE of the leaves of this species exhibited a moderate ACE-inhibitory activity. Chlorogenic acid, orientin, isoorientin, catechin, epicatechin, protocatechuic acid, and procyanidin B2 and C1 were identified through bioguided fractionation (Lacaille-Dubois et al. 2001; Li et al. 2013). Of these compounds, the most effective were isoorientin (48 ± 1%) and procyanidin C_1_ (45 ± 2%) at 0.33 mg/mL. Meanwhile the other compounds showed low activities (between 4-25%) at the same concentration. It is suggested that the mixture works synergistically causing the total effect of EtE (40 ± 4%) (Lacaille-Dubois et al. 2001).

## Selection of chemical markers for the genus *Cecropia*

The chemical markers are ‘chemically defined constituents or groups of constituents of a herbal substance, a herbal preparation or a herbal medicinal product which serve for quality control purposes, independent of whether they have any therapeutic activity’. EMA describes two different categories of markers. The constituents of an herbal medicine responsible of its therapeutic activity or active markers; and the constituents that are characteristics of its taxon or analytical markers (European Medicines Agency [EMA] 2008).

In order to determine the most appropriate chemical markers for the quality control of *Cecropia* sp., we have used the Herbal Chemical Marker Ranking System (Herb MaRS) developed by The National Institute of Complementary Medicine (NICM) at the University of Western Sydney, 2014 (Bensoussan et al. 2015). Herb MaRS approach considers different factors related to the plant constituents, such as the availability of bioactivity studies and pure chemical reference standards; relationship of the traditional or current use of the herb to its therapeutic application or the pharmacological effects, concentration of the chemical marker in the herbal product, and toxicity or maximum recommended dose.

Herb MaRS criteria provide a prioritized list of chemical markers rationally ranked using a scale from 0 to 5, where 5 indicates the most suitable chemical marker. A rank of 0 designates the least suitable. Additionally, the category of ‘X’ denotes a lack of bioactivity studies on the compound during the selection.

The relevant compounds that have been identified in five *Cecropia* sp. and that we have selected are shown in Table 1, along with their biological activities and priority ranking.

**Table 1.** Compounds in *Cecropia* sp. with Herb Mars score on potential biological activities.

Compound	Plant species	Plant part	Concentration	Biological activity	Ranking score	Standard available	References
Phenolic acids							
Chlorogenic acid	*C. obtusifolia*	Leaves	DL↑, AE↑, BuE ↑	Anti-diabetic	5	Y	(Andrade-Cetto & Wiedenfeld 2001; Andrade-Cetto & Heinrich 2005; Revilla-Monsalve et al. 2007; Alonso-Castro et al. 2008; Andrade-Cetto et al. 2008)
	*C. obtusifolia y C. peltata*	Leaves	ME↑	Anti-diabetic	5	Y	(Nicasio et al. 2005; Andrade-Cetto et al. 2007)
	*C. glaziovii*	Leaves	AE↑	Anti-inflammatory, anti-hypertensive	5	Y	(Tanae et al. 2007; Costa et al. 2011a; Müller et al. 2016)
	*C. pachystachya*	Leaves	DL↑, ME↑, AE↑	Anti-diabetic, anti-inflammatory, wound healing, leishmanicidal, neuroprotective, QS inhibitor	5	Y	(Aragão et al. 2010; Costa et al. 2011a; Cruz et al. 2013; Gazal et al. 2014; Maquiaveli et al. 2014; Pacheco et al. 2014; Gazal et al. 2015; Duque et al. 2016; Ortmann et al. 2016)
	*C. hololeuca*	Leaves	NR	Anti-hypertensive	1	Y	(Lacaille-Dubois et al. 2001)
Caffeic acid	*C. glaziovii*	Leaves	EtE↓	NR	1	Y	(Arend et al. 2011)
Vanillic acid	*C. obtusifolia*	Leaves	DCME↑	Anti-inflammatory	0	Y	(Guerrero et al. 2010; Kim et al. 2011)
Flavonoids							
Isoorientin	*C. obtusifolia*	Leaves	DL↑, AE↑, BuE↑	Anti-diabetic	5	Y	(Andrade-Cetto & Wiedenfeld 2001; Revilla-Monsalve et al. 2007; Andrade-Cetto et al. 2008)
	*C. peltata*	Leaves	NR	Anti-diabetic	1	Y	(Andrade-Cetto et al. 2007)
	*C. glaziovii*	Leaves	AE↑	Anti-inflammatory, anti-hypertensive, anti-ulcer and anti-secretory gastric activities	5	Y	(Tanae et al. 2007; Souccar et al. 2008; Costa et al. 2014; Müller et al. 2016)
		Stipules	NR	Anti-hypertensive	1	Y	(Lacaille-Dubois et al. 2001)
	*C. pachystachya*	Leaves	DL↑, ME↑, AE↑	Anti-diabetic, anti-inflammatory, anti-depressant, wound healing, neuroprotective, QS inhibitor	5	Y	(Oliveira et al. 2003; Chanmahasathien et al. 2004; Aragão et al. 2010; Costa et al. 2011a; Brango-Vanegas et al. 2014; Gazal et al. 2014; Pacheco et al. 2014; Gazal et al. 2015; Duque et al. 2016; Ortmann et al. 2016)
	*C. hololeuca*	Leaves	NR	Anti-hypertensive	1	Y	(Lacaille-Dubois et al. 2001)
Orientin	*C. glaziovii*	Leaves	AE↑	Anti-hypertensive, anti-ulcer and anti-secretory gastric activities	5	Y	(Tanae et al. 2007; Souccar et al. 2008)
	*C. pachystachya*	Leaves	DL↑, ME↑, AE↑	Anti-diabetic, anti-inflammatory, anti-depressant, wound healing, leishmanicidal, neuroprotective, QS inhibitor	5	Y	(Chanmahasathien et al. 2004; Aragão et al. 2010; Costa et al. 2011a; Cruz et al. 2013; Brango-Vanegas et al. 2014; Gazal et al. 2014; Maquiaveli et al. 2014; Pacheco et al. 2014; Gazal et al. 2015; Duque et al. 2016; Ortmann et al. 2016)
	*C. hololeuca*	Leaves	NR	Anti-hypertensive	1	Y	(Lacaille-Dubois et al. 2001)
Isovitexin	*C. glaziovii*	Leaves	AE↑	Anti-inflammatory, anti-hypertensive, anti-ulcer and anti-secretory gastric activities	5	Y	(Tanae et al. 2007; Souccar et al. 2008; Costa et al. 2011a; 2014)
	*C. pachystachya*	Leaves	ME↑ AE↑	Anti-depressant, leishmanicidal, neuroprotective, QS inhibitor	5	Y	(Lacaille-Dubois et al. 2001; Costa et al. 2011a; Brango-Vanegas et al. 2014; Gazal et al. 2014; 2015; Ortmann et al. 2016)
Vitexin	*C. pachystachya*	Leaves	DL↓ ME↓	Anti-inflammatory, QS inhibitor	3	Y	(Chanmahasathien et al. 2004; Brango-Vanegas et al. 2014)
Rutin	*C. pachystachya*	Leaves	DL↓ ME↑	Anti-inflammatory, QS inhibitor	5	Y	(Chanmahasathien et al. 2004; Brango-Vanegas et al. 2014)
Isoquercitrin	*C. glaziovii*	Stipules and leaves	NR	Anti-hypertensive	1	Y	(Lacaille-Dubois et al. 2001)
	*C. pachystachya*	Leaves	NR	Leishmanicidal, neuroprotective, anti-depressant	1	Y	(Lacaille-Dubois et al. 2001; Cruz et al. 2013; Gazal et al. 2014; 2015; Ortmann et al. 2016)
Quercetin	*C. pachystachya*	Leaves	NR	Leishmanicidal	1	Y	(Cruz et al. 2013)
Apigenin	*C. pachystachya*	Leaves	NR	Leishmanicidal	1	Y	(Cruz et al. 2013)
Apigenin-6-galactosyl-6′′-O-galactopyranoside	*C. pachystachya*	Leaves	DL↓	NR	0	N	(Oliveira et al. 2003)
Luteolin	*C. pachystachya*	Leaves	NR	Leishmanicidal	1	Y	(Cruz et al. 2013)
Catechin	*C. glaziovii*	Leaves	AE ↑ (Tanae et al. 2007)	Anti-hypertensive, anti-depressant-like effect, anti-ulcer and anti-secretory gastric activities	5	Y	(Rocha et al. 2007; Tanae et al. 2007; Souccar et al. 2008)
		Stipules	NR	Anti-hypertensive	1	Y	(Lacaille-Dubois et al. 2001)
	*C. pachystachya*	Leaves	NR	Anti-hypertensive, leishmanicidal	1	Y	(Lacaille-Dubois et al. 2001; Cruz et al. 2013)
	*C. hololeuca*	Leaves	NR	Anti-hypertensive	1	Y	(Lacaille-Dubois et al. 2001)
Epicatechin	*C. glaziovii*	Leaves	AE ↑	Anti-hypertensive, anti-ulcer and anti-secretory gastric activities	5	Y	(Tanae et al. 2007; Souccar et al. 2008)
		Stipules	NR	Anti-hypertensive	1	Y	(Lacaille-Dubois et al. 2001)
	*C. pachystachya*	Leaves	NR	Anti-hypertensive, leishmanicidal	1	Y	(Lacaille-Dubois et al. 2001; Cruz et al. 2013)
	*C. hololeuca*	Leaves	NR	Anti-hypertensive	1	Y	(Lacaille-Dubois et al. 2001)
Procyanidin B2	*C. glaziovii*	Leaves	AE ↑	Anti-hypertensive, anti-depressant-like effect, anti-ulcer and anti-secretory gastric activities	5	Y	(Rocha et al. 2007; Tanae et al. 2007; Souccar et al. 2008)
		Stipules	NR	Anti-hypertensive	1	Y	(Lacaille-Dubois et al. 2001)
	*C. pachystachya*	Leaves	NR	Anti-hypertensive	1	Y	(Lacaille-Dubois et al. 2001)
	*C. hololeuca*	Leaves	NR	Anti-hypertensive	1	Y	(Lacaille-Dubois et al. 2001)
Procyanidin B3 isomer	*C. glaziovii*	Leaves	AE↑	Anti-hypertensive, anti-depressant-like effect, anti-ulcer and anti-secretory gastric activities	0	N	(Rocha et al. 2007; Tanae et al. 2007; Souccar et al. 2008)
Procyanidin B5	*C. glaziovii*	Leaves	AE↑	Anti-hypertensive, anti-ulcer and anti-secretory gastric activities	5	Y	(Tanae et al. 2007; Souccar et al. 2008)
Procyanidin C1	*C. glaziovii*	Leaves	AE↑	Anti-hypertensive, anti-ulcer and anti-secretory gastric activities	5	Y	(Tanae et al. 2007; Souccar et al. 2008)
		Stipules	NR	Anti-hypertensive	1	Y	(Lacaille-Dubois et al. 2001)
	*C. hololeuca*	Leaves	NR	Anti-hypertensive	1	Y	(Lacaille-Dubois et al. 2001)
Protocatechuic acid	*C. pachystachya*	Leaves	NR	NR	1	Y	(Lacaille-Dubois et al. 2001)
	*C. hololeuca*	Leaves	NR	Anti-hypertensive	1	Y	(Lacaille-Dubois et al. 2001)
Terpenic and steroidal compounds							
2-α-Acetoxy-3β,19α -dihydroxy-11α,12α -epoxy-ursan-28,13β-olide	*C. pachystachya*	Roots	DL↓	NR	0	N	(Machado et al. 2008)
2-α-Acetyl tormentic acid	*C. pachystachya*	Roots	NR	Cytotoxic	0	N	(Rocha et al. 2007, 2012a)
2-*O*-Acetyl euscaphic acid	*C. pachystachya*	Roots	DL↑	NR	0	N	(Machado et al. 2008)
3-β-Acetoxy-2α,19α-dihydroxy-11α,12α-epoxy-ursan-28,13β-olide	*C. pachystachya*	Roots	DL↓	NR	0	N	(Machado et al. 2008)
3-β-Acetyl tormentic acid	*C. pachystachya*	Roots	DL↓	Cytotoxic	0	N	(Oliveira et al. 2005; Rocha et al. 2007, 2012a)
4,22-Cholestadien-3-one	*C. obtusifolia*	Leaves	DCME↑	NR	0	N	(Guerrero et al. 2010)
4-Cholestene-3,24-dione	*C. obtusifolia*	Leaves	DCME↑	NR	0	N	(Guerrero et al. 2010)
α-Amyrin	*C. pachystachya*	Leaves	DL↑, DCME↑	Anti-inflammatory	5	Y	(Schinella et al. 2008)
β-Sitosterol	*C. obtusifolia*	Leaves	NR	Anti-inflammatory	1	Y	(Andrade-Cetto & Heinrich 2005; Loizou et al. 2010)
	*C. pachystachya*	Leaves	DL↑, DCME↑	Anti-inflammatory	5	Y	(Hikawczuk et al. 1998; Schinella et al. 2008)
Euscaphic acid	*C. pachystachya*	Roots	DL↓	Cytotoxic	0	Y	(Oliveira et al. 2005; Rocha et al. 2007, 2012a)
Isoarjulonic acid	*C. pachystachya*	Roots	DL↓	NR	0	N	(Oliveira et al. 2005)
Oleanolic acid	*C. pachystachya*	Leaves	NR	Anti-inflammatory	1	Y	(Hikawczuk et al. 1998)
Pomolic acid	*C. pachystachya*	Leaves	DL↑ DCME↑	Anti-inflammatory	5	Y	(Schinella et al. 2008)
Stigmast-4-en-3-one	*C. obtusifolia*	Leaves	DCME↑	Anti-inflammatory, anti-diabetic	1	Y	(Jamaluddin et al. 1995; Alexander-Lindo et al. 2004; Guerrero et al. 2010; Tewtrakul et al. 2010)
Stigmasterol	*C. obtusifolia*	Leaves	NR	Anti-inflammatory	1	Y	(Andrade-Cetto & Heinrich 2005; Gabay et al. 2010)
Tormentic acid	*C. pachystachya*	Root-wood	DL↑ EtE↑	Anti-inflammatory, anti-malaric and cytotoxic	5	Y	(Oliveira et al. 2005; Rocha et al. 2007, 2012a; Uchoa et al. 2010)
Ursolic acid	*C. pachystachya*	Leaves	DL↑ DCME↑	Anti-inflammatory	5	Y	(Schinella et al. 2008)
Anthraquinones							
Aloe-emodin	*C. obtusifolia*	Leaves	NR	Anti-inflammatory	1	Y	(Choi et al. 2013; Yan et al. 2013; Kshirsagar et al. 2014; Park et al. 2016)
Chrysophanol	*C. obtusifolia*	Leaves	NR	Anti-inflammatory	1	Y	(Choi et al. 2013; Yan et al. 2013; Kshirsagar et al. 2014; Park et al. 2016)
Emodin	*C. obtusifolia*	Leaves	NR	Anti-inflammatory	1	Y	(Choi et al. 2013; Yan et al. 2013; Kshirsagar et al. 2014; Park et al. 2016)
Physcion	*C. obtusifolia*	Leaves	NR	Anti-inflammatory	1	Y	(Choi et al. 2013; Yan et al. 2013; Kshirsagar et al. 2014; Park et al. 2016)
Rehin	*C. obtusifolia*	Leaves	NR	Anti-inflammatory	1	Y	(Choi et al. 2013; Yan et al. 2013; Kshirsagar et al. 2014; Park et al. 2016)
Other compounds							
1-(2-Methyl-1-nonen-8-il)-aziridine	*C. obtusifolia*	Leaves	NR	NR	0	N	(Andrade-Cetto & Heinrich 2005)
2-Methylbenzaldehyde	*C. obtusifolia*	Leaves	DCME↑	NR	0	N	(Guerrero et al. 2010)
2,3-Dihydrobenzofuran	*C. obtusifolia*	Leaves	DCME↑	NR	0	Y	(Guerrero et al. 2010)
3′-Methoxyacetophenone	*C. obtusifolia*	Leaves	DCME↑	NR	0	Y	(Guerrero et al. 2010)
4-Ethyl-5-(n-3valeroil)-6-hexahy- drocoumarin	*C. obtusifolia*	Leaves	NR	NR	0	N	(Andrade-Cetto & Heinrich 2005)
4-Vinyl-2-methoxy-phenol	*C. obtusifolia*	Leaves	DCME↑	NR	0	Y	(Guerrero et al. 2010)
Palmitic acid	*C. obtusifolia*	Leaves	DCME↑	Anti-inflammatory	1	Y	(Guerrero et al. 2010; Aparna et al. 2012)
Stearic acid	*C. obtusifolia*	Leaves	DCME↑	Anti-inflammatory	1	Y	(Guerrero et al. 2010; Pan et al. 2010)
*trans*-Phytol	*C. pachystachya*	Leaves	DL↑, DCME↑	Anti-inflammatory	5	Y	(Schinella et al. 2008)

DL: Dried material; AE: aqueous extract; BuE: butanolic extract; ME: methanolic extract; DCME: dichloromethane extract; HE: hexane extract; NR Not reported. Too low concentration (↓↓): <5 μg/g in the herb or finished product. Low concentration (↓): 5-50 μg/g in the herb or finished product. Relative high concentration (↑): >50 μg/g in the herb or finished product..

### *Cecropia obtusifolia* Bertol

Chlorogenic acid (a cinnamic acid derivative) and isoorientin (a flavonoid C-glycoside) are the major compounds identified from leaves. Their relative high concentration (>50 μg/g, DL), hypoglycemic, anti-inflammatory effect, and analytical standard commercially available make them appropriate to be selected as active markers of choice for the analysis of *C. obtusifolia* as medicinal plant. Both analytes were ranked with a score of 5.

Anthraquinones, palmitic, stearic, and vanillic acids received a score of 1, as their relative concentration (DL) remains uncertain for this species. All of these compounds are available from commercial suppliers. The rest of reported compounds for this species received a score of 0 as there are no bioactivity studies currently available related to its traditional use (see Table 1).

### *Cecropia peltata* L

Chlorogenic acid and isoorientin were chosen as markers for quality evaluation. The former received a score of 5 due to its correlation with the hypoglycemic effect produced by AE, BuE, and ME (Nicasio et al. 2005; Andrade-Cetto et al. 2007). It is suggested, similarly to *C. obtusifolia*, that isoorientin content may support the antidiabetic effect of this plant (Andrade-Cetto et al. 2007). Isoorientin was scored 1 because there is no evidence of its concentration in the plant material.

### *Cecropia glaziovii* Snethl

Several pharmacological activities were associated to its standardized AE and BuF, the latter rich in flavonoids, procyanidins, catechins, and phenolic acids. Chlorogenic acid, isovitexin, isoorientin, orientin, catechin, epicatechin, and procyanidins B2, B5 and C1 were selected as chemical markers and received a score of 5 due to their pharmacological importance, relative high concentration (>50 μg/g) in the aqueous extract (Costa et al. 2014), good chemical stability, and analytical standards commercially available. Isoquercetrin and caffeic acid (Arend et al. 2011; Costa et al. 2014) received a scoring of 1 because there is no evidence of their concentrations in the plant material and activity was not related to the traditional use of the herb, respectively. Procyanidin B3 isomer is ranked as low in importance since no pure reference standard was commercially available.

### *Cecropia pachystachya* Trécul

Chlorogenic acid, orientin, isoorientin, isovitexin, rutin, β-sitosterol, α-amyrin, *trans*-phytol, pomolic, ursolic, and tormentic acids were selected as suitable analytes for monitoring and received a score of 5 due to their pharmacological activities, relative high concentration (>50 μg/g), good chemical stability, and commercial availability of analytical standards. Vitexin was scored with a 3 due to its low relative concentration in the plant material.

In addition, isoquercetrin, quercetin, apigenin, luteolin, catechin, epicatechin, procyanidin B2, protocatechuic, and oleanolic acids received a score of 1 since there is no available information about their relative concentration in the plant material. Apigenin-6-galactosyl-6″-*O*-galactopyranoside has low importance because no standard is commercially available.

We assigned a score of 0 to euscaphic, 2-*O*-acetyl euscaphic, 2-α-acetyl tormentic, 3-β-acetyl tormentic, isoarjunolic acids, 2-α-acetoxy-3β,19α-dihydroxy-11α,12α-epoxy-ursan-28,13β-olide, and 3-β-Acetoxy-2α,19α-dihydroxy-11α,12α-epoxy-ursan-28,13β-olide because there is no evidence on the concentrations of these compounds in the leaves or wood of this plant, which are traditionally used as medicine.

### *Cecropia hololeuca* Miq

All compounds described for this species were scored 1 since there is no evidence on their concentrations in the plant material.

## Conclusions

Insufficient information is available about the chemical constituents of most medicinal plants for guaranteeing their quality, safety, and efficacy. Therefore, it is necessary to establish comprehensive standards for assessing the quality of herbal drugs. Due to the complexity of phytomedicines, only a small group of compounds is chosen for quality purposes. Chemical and pharmacological studies represent useful tools for the selection of chemical markers for addressing the quality evaluation of medicinal plants.

In this review we presented the most extensively studied species of the *Cecropia* genus, which are traditionally used as medicine in Latin America. *C. obtusifolia* and *C. peltata* are renowned for their hypoglycaemic activity, while *C. glaziovii*, *C. pachystachya* and *C. hololeuca* are frequently used for the treatment of inflammation, hypertension, and respiratory conditions. The latter two species are also well known as anticancer agents. The medicinal use of almost all of these species is officially recognized in Mexico (*C. obtusifolia*), Argentina (*C. pachystachya*), and Brazil (*C. glaziovii* and *C. hololeuca*) through their National Pharmacopoeias and Formularies.

**Figure 1.**
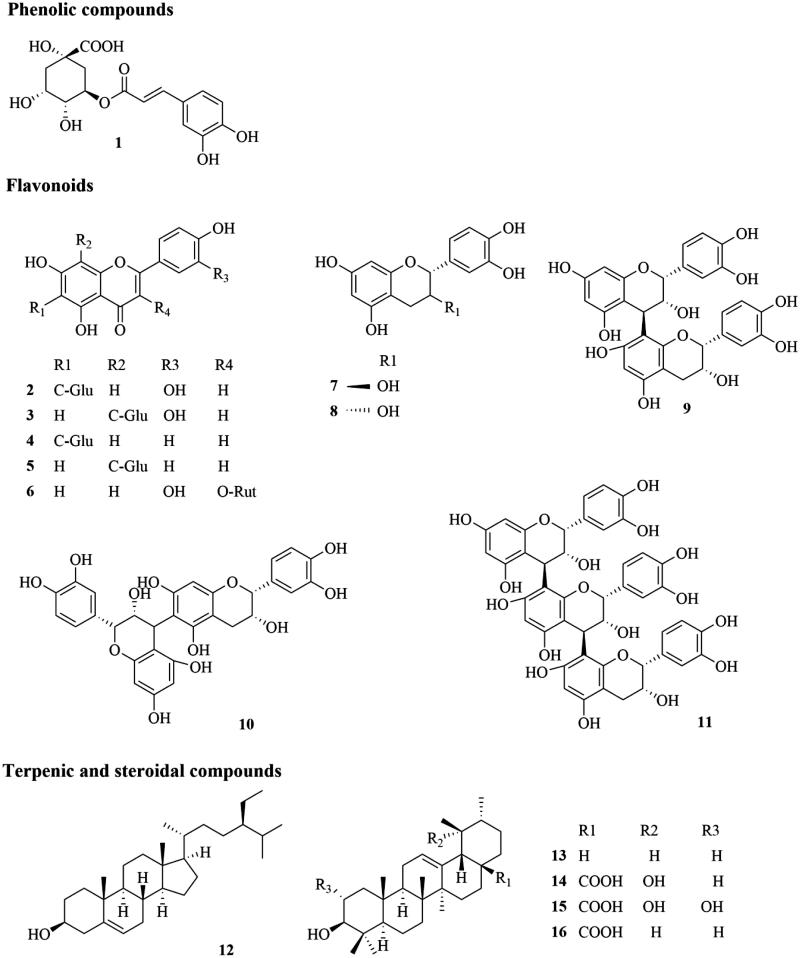
Chemical structures of selected markers: chlorogenic acid (**1**), isoorientin (**2**), orientin (**3**), isovitexin (**4**), vitexin (**5**), rutin (**6**), catechin (**7**), epicatechin (**8**), procyanidin B2 (**9**), procyanidin B5 (**10**), procyanidin C1 (**11**), β-sitosterol (**12**), α-amyrin (**13**), pomolic acid (**14**), tormentic acid (**15**), ursolic acid (**16**).

Chlorogenic acid, glycosidic flavonoids (orientin, isoorientin, vitexin, isovitexin, and rutin), catechin, epicatechin, procyanidins (B2, B5, and C1), steroids (β-sitosterol), and triterpenoids (α-amyrin, pomolic, tormentic, and ursolic acids) (Figure 1) have been chosen as chemical markers for the quality evaluation of leaves according to the ranking score of Herb MaRS. The biological activities of these compounds have been related to their traditional uses. The role of chlorogenic acid and isoorientin in the hypoglycaemic effect of *C. obtusifolia* and *C. peltata* has been well established. Similarly, the anti-hypertensive and anti-inflammatory activities of *C. glaziovii* and C. *pachystachya* have been correlated to the presence of flavonoids, cathechins, proanthocyanidins, terpenic, and steroidal compounds. Additionally, these analytes have been identified as the main components of the plant material (with relative concentrations above 5 μg/g). Suitable reference standards are commercially available and they can be easily detected with current technology.

Most of these analytes have previously been selected as chemical markers for the qualitative and quantitative assessment of a number of herbal drugs described in the European Pharmacopoeia (Ph. Eur.) (Council of Europe 2014). For example, chlorogenic acid is used as a marker compound in the nettle leaf (*Urtica dioica*, *Urtica urens*, or a mixture of the two species) and artichoke leaf (*Cynara scolymus*) monographs. Orientin, isoorientin, isovitexin, vitexin, and rutin, for their part, serve as analytes in the passion flower (*Passiflora incarnata*) monograph. Other chemical markers, including catechin, procyanidins, β-sitosterol, α-amyrin, and ursolic acid, are described in the bistort rhizome (*Persicaria bistorta*), hawthorn berry (*Crataegus monogyna*), saw palmetto fruit (*Serenoa repens*), and *Pygeum africanum* bark (*Prunus africana*) monographs.

Some secondary metabolites, such as chlorogenic acid, orientin, isoorientin, vitexin, isovitexin, and catechin, are reported in all of these different species, including *C. obtusifolia*, *C. peltata*, *C. glaziovii*, and *C. pachystachya*. An HPLC method developed for the quantification of the main phenolic compounds from leaves showed significant differences between *pachystachya* and *glaziovii* species (Costa et al. 2011a). We suggest that more studies, both chemical and pharmacological ones, need to be performed using the chemical markers that we propose, for the development of analytical methods and monographs for both qualitative and quantitative evaluations. Fingerprinting profiles of the marker compounds may be very helpful for comparing similarities and differences between the species. More statistical studies about seasonal, phenotypic, and demographical variables may be taken into consideration as well.
